# Performance evaluation of typical automatic technical safety barriers in chemical atmospheric storage tank areas

**DOI:** 10.1371/journal.pone.0340102

**Published:** 2026-02-11

**Authors:** Tianyu Wang, Mingguang Zhang, Xueliang Tan

**Affiliations:** 1 College of Safety Science and Engineering, Nanjing Tech University, Nanjing, Jiangsu, China; 2 Jiangsu Key Laboratory of Hazardous Chemicals Safety and Control, Nanjing, Jiangsu, China; Henan Polytechnic University, CHINA

## Abstract

Atmospheric storage tank farms in chemical industrial parks pose significant safety risks due to their complex layouts and the storage of flammable and explosive substances, which can trigger domino accidents. Automated safety barriers are widely applied, but their quantitative performance assessment remains a challenge in frameworks such as Accidental Risk Assessment Methodology for Industries (ARAMIS). To address this gap, this study proposes a quantitative model that evaluates safety barriers through the combined metrics of effectiveness and reliability. Water spray systems were selected as a representative barrier and analyzed using Pyrosim and ANSYS Workbench. The simulations revealed that tank failures mainly occur at the junction of the tank wall and roof as material properties degrade at elevated temperatures. Results showed that while water spray systems reduce thermal radiation damage, their protective effect decreases with increasing wind speed. Effectiveness was quantified through failure time extension and failure probability reduction, whereas reliability was evaluated via a Bayesian Network (BN) model. Integrating these factors produced a comprehensive performance index. Application of the model in a case study yielded a performance score of 0.752 for the water spray system, demonstrating both its protective capability and the practicality of the proposed method. Compared with the traditional ARAMIS framework, this approach offers improved precision and a more robust quantitative assessment for safety barrier performance in chemical storage tank areas.

## 1. Introduction

With the rapid development of China’s social economy, the chemical industry has gradually become a pillar of the national economy, and its industrial scale has risen to the largest in the world. To optimize resource allocation and maximize economic benefits, the large-scale and intensive development of petrochemical storage facilities has become an inevitable trend. In China’s oil and gas storage areas, vertical cylindrical steel storage tanks are the most widely used type [1]. However, due to the dense layout of storage tanks, the complexity of pipeline systems, and the large quantities of hazardous chemicals involved—mostly flammable, explosive, and toxic substances—domino accidents are highly likely to occur, potentially leading to secondary or cascading major accidents, as demonstrated by several real-world events. For example, on June 2, 2013, an explosion occurred in a miscellaneous material tank during hot work in the tank area of PetroChina Dalian Petrochemical Company [2], resulting in leaks and subsequent fires and explosions in three nearby tanks. The accident caused four fatalities and direct economic losses of approximately 6.97 million yuan. More recently, on August 6, 2022, a large oil tank at a depot in Matanzas Province, Cuba, was struck by lightning, triggering a fire that led to a series of combustion and explosion accidents, resulting in two deaths and 132 injuries [3].

After an initial accident (leakage, fire, or explosion) occurs in a storage tank area, consequences such as thermal radiation and shock waves act on adjacent tanks, triggering secondary accidents and leading to accident escalation and expansion, a process with significant multi-hazard coupling effects [4]. In large-scale storage tank areas, the use of safety barriers is an effective means to control accident escalation and mitigate severe consequences. Haddon [5], based on the energy model and ten disaster prevention and control strategies in 1980, pointed out that setting up protective barriers in the path of energy transmission to isolate humans from energy marked the origin of safety barrier research. Kjellén et al. [6], from the perspective of evolutionary processes, divided accidents into initiation, termination, and harm stages, considering safety barriers as the main protective measures to hinder the occurrence, escalation, and evolution of accidents. Svenson [7] regarded safety barriers as “functions” that interrupt the accident evolution chain, emphasizing that any accident stems from a series of failures and the continuous failure of all barrier functions. Duijm et al. [8] defined them as systems that prevent, limit, or mitigate the release of hazards, including both preventive systems that reduce the probability of accidents and mitigation systems that reduce consequences. DNV GL [9] considered safety barriers as measures to prevent hazardous events, which can include multiple technical, operational, and organizational elements and perform one or more safety functions, determining the purpose of the barriers. Safety barriers are defined in the ARAMIS methodology as technical, organizational, or human measures designed to prevent the initiation of major accidents or to mitigate their consequences [10]. Within this framework, barriers are classified into preventive and protective types. In the context of atmospheric storage tank farms, protective technical safety barriers—such as water deluge systems, sprinklers, or water curtains—are essential in mitigating domino effects triggered by pool fires.

However, ARAMIS has been criticized for relying heavily on qualitative or semi-quantitative judgments, which limits its precision in assessing barrier performance under dynamic conditions such as varying wind speeds. Thus, the central research question of this study is: How can the performance of water-based protective barriers in chemical storage tank farms be quantitatively evaluated by integrating effectiveness and reliability? Addressing this question is critical for improving risk assessments of domino accidents and providing decision-makers with more accurate guidance on safety system design.

Many scholars have established some failure laws and models for chemical storage tanks under scenarios such as fires and explosions. However, due to the strict requirements for large-scale pool fire experiments, most studies are based on numerical simulations and small-scale experiments. Cozzani et al. [11,12] conducted numerical simulation studies on container failure under a single accident scenario, focusing on the tolerance characteristics of atmospheric and pressure vessels under different thermal radiation intensities. Iannaccone et al. [13], in their research on the thermal response process of liquefied natural gas storage tanks, found that under low filling rate conditions, the increase in gasification space leads to a sharp rise in tank pressure, which significantly shortens the time from heating to structural failure of the container. Zhou et al. [14] proposed an improved Probit model, introducing the concept of “heat dose” to quantitatively analyze equipment failure time under multi-source fires, considering the dynamic evolution and synergistic effect of fires, which improves the accuracy of risk assessment for fire-induced multi-level domino effects. Ding et al. [15] proposed an equipment vulnerability model (FESEM) that considers the temporal and spatial synergistic effect of fire thermal radiation and explosion overpressure. By studying the temperature rise and strength decline of storage tanks under fire and the equivalent stress caused by explosions, it quantitatively evaluates equipment failure time and accident escalation probability, thus providing a more accurate and mechanistic method for risk assessment of domino effects under multi-hazard coupling in chemical plants. However, in reality, relevant environmental factors significantly promote the development of accidents in fire scenarios and affect the function of some safety barriers, which most scholars have not yet considered in depth.

The performance evaluation of safety barriers aims to measure the protective effect of single or multiple safety barriers on protected targets. Currently, many studies are based on subjective qualitative or semi-quantitative methods such as expert scoring, which easily leads to inaccurate results in safety barrier performance evaluation. Among them, Landucci et al. [16,17] proposed a method based on Layer of Protection Analysis (LOPA) to define and quantify the performance of safety barriers in preventing accident escalation, obtain data on common types of barriers to describe their effectiveness and failure probability at critical moments, and also analyze the impact of safety protection measures on fire-induced domino effects and evaluate the effectiveness of safety protection devices. Prashanth et al. [18] studied the factors affecting the failure of safety barriers in onshore natural gas drilling operations, identified 25 key influencing factors, verified them through professional seminars, and adopted a scoring mechanism to evaluate the performance of safety barriers, so as to quantify the impact of each factor on barrier functions. Misuri et al. [19] proposed a method to assess the performance changes of safety barriers in Na-Tech accidents, which quantifies the impact of natural factors (such as floods and earthquakes) on the probability and frequency of secondary accident scenarios by considering their effects on safety barriers. Maio F et al. [20] transformed the identification of optimal performance parameters of safety barriers into a multi-objective optimization (MOO) problem. Taking storage tank facilities filled with flammable substances as a case study, they studied the safety protection effect in Na-Tech accident scenarios, configured six types of safety barriers (including active, passive, and procedural barriers), and used a phenomenological dynamic model to evaluate their performance, so as to optimize the accident protection effect. Remennikov et al. [21] evaluated the protective performance of steel-concrete (SC) composite barriers under ultra-high-speed impact of explosive fragments (EFP) through experiments and numerical simulations, and the study revealed the influence mechanism of the relative thickness ratio of steel plates to concrete on the anti-explosion performance of protective structures. Azarmehri et al. [22] systematically reviewed various methods for assessing the impact of human and organizational factors (HOFs) on the performance of safety barriers, emphasized their key role in risk management of the process industry, and pointed out that the integration of fuzzy logic and Bayesian networks can effectively address the uncertainty of expert judgments and multi-factor interaction issues. Yuan S et al. [23,24] proposed a method integrating event tree analysis, computational fluid dynamics simulation, and evacuation modeling to evaluate the performance and synergistic effect of technical safety barriers and emergency evacuation in toxic gas leakage accidents in chemical plants. Meanwhile, based on efficiency and cost, they proposed a method to optimize the maintenance cost of safety barriers using the Bow-tie method, simulation, and genetic algorithm (GA). Wu et al. [25] proposed a hybrid method based on DBN (Dynamic Bayesian Network) to analyze the dynamic performance of safety barriers. This method comprehensively evaluates the dynamic performance of safety barriers from four aspects: preventive maintenance, imperfect maintenance, degradation effect, process requirements, and maintenance cost. Wu et al. [26] studied the protective effect of water spray systems on pool fire thermal radiation through numerical simulations, and the simulations showed that the heat flux mitigation coefficient of the protective device is 0.2–0.3, and the protective device prolongs the tank failure time by 5–20 minutes.

Although the ARAMIS framework has been widely adopted in Europe for safety barrier assessment [8], it has been criticized for several shortcomings. First, its evaluation of barrier effectiveness relies heavily on expert judgment and lacks quantitative simulation support [16,19]. Second, its treatment of response time is highly uncertain in practical applications, as it does not sufficiently account for environmental or operational variability [18]. Finally, ARAMIS tends to provide static assessments and cannot easily capture the dynamic degradation of barrier performance over time [25]. These limitations reduce its precision in quantitative risk assessment of chemical storage tank scenarios.

This study has advanced the ARAMIS safety barrier evaluation system. It achieved this by establishing a method for quantitative performance assessment, which is based on effectiveness and reliability metrics derived from extensive data simulations. This approach successfully overcomes the limitations of the original ARAMIS methodology. Models of atmospheric storage tanks under thermal radiation in various environmental wind scenarios have been developed. This was accomplished using numerical simulation software, specifically for tanks protected by typical automated technical safety barriers. The failure processes of these tanks were investigated. Based on the observed failure times, failure time models were constructed for these tanks under thermal radiation with barrier protection. These models served as the foundational basis for analyzing barrier effectiveness. Concurrently, barrier reliability was derived through Bayesian network analysis. Finally, by integrating effectiveness and reliability, a comprehensive performance score was obtained, enabling a precise quantitative assessment of safety barriers.

Compared with the traditional ARAMIS framework, our model addresses these shortcomings by incorporating numerical simulation to quantify failure time and thermal radiation effects, thereby reducing reliance on expert judgment, explicitly modeling environmental wind factors, which ARAMIS does not account for, and integrating reliability analysis via Bayesian networks, enabling a dynamic and probabilistic evaluation instead of static assumptions. Similar approaches have been proposed in recent works, confirming the importance of supplementing ARAMIS with quantitative methodologies.

The following is a detailed description of the research content in this paper: In Section 2, simulation models of atmospheric storage tanks under thermal radiation from pool fire flames are established using Pyrosim and ANSYS Workbench software combined with accident statistics. In Section 3, water spray systems are selected as typical automatic technical safety barriers. The influence of environmental wind factors on the protective effect of water spray systems and the failure criteria of storage tanks with and without water spray systems are considered. In Section 4, computational models for the effectiveness and reliability of water spray systems are constructed based on simulation results and Bayesian networks. The performance level of water spray systems in a chemical atmospheric tank area is calculated through practical cases. Discussion are drawn in Section 5. Conclusions are drawn in Section 6.

## 2. Establishment of the numerical model

A statistical analysis of 138 atmospheric storage tank accidents between 1970 and 2024 revealed that gasoline was the most common medium involved, accounting for 23.9% of incidents (Table 1). The most frequent accident chain expansion was “fire → fire” (41.7%) (Table 2). Therefore, water spray systems were selected as the safety barrier for this study. The accident data were categorized by accident medium and escalation type, and percentage values were calculated as ratios of the total 138 cases.

**Table 1:** Statistics of the initial accident media type.

Initial accident medium	Number of accidents	Ratio(%)	Initial accident medium	Number of accidents	Ratio(%)
Gasoline	33	23.9	Heavy oil	2	1.4
Crude oil	20	14.5	Solvent oil	2	1.4
Explosive volatile gas	16	11.6	Aromatics	1	0.7
Naphtha	9	6.5	Light oil	1	0.7
Diesel oil	9	6.5	Hydrogen peroxide	1	0.7
Dirty oil	5	3.6	Isopropanol	1	0.7
Fuel oil	5	3.6	Liquid wax	1	0.7
Alkanes	4	3.0	Iron sulfide	1	0.7

**Table 2:** Statistics of the accident consequences extended form.

Consequence expansion form	Quantity	Ratio
Fire→Fire	60	0.417
Fire→Blast	36	0.250
Blast→Fire	32	0.222
Fire→Leak	5	0.035
Blast→Blast	4	0.028
Blast→Leak	3	0.021
Leak→Blast	2	0.014
Leak→Fire	2	0.014

### 2.1. Pool fire thermal radiation calculation model

#### 2.1.1. Pool fire model.

The combustion medium is gasoline, and the specific thermophysical parameters are shown in Table 5. The calculation formulas proposed by Thomas [27] are presented in Equations 2-1–2-3. The formula for calculating the mass burning rate of flammable liquids, proposed by Babrauskas [28], is shown in Equation 2-4.

**Table 3:** Basis data of storage tanks with different volumes.

Volume (m³)	Diameter (m)	Height (m)	Curvature radius (m)
1000	11.5	11.9	13.8
2000	15.8	13.1	18.9
3000	18.9	13.8	21.6
5000	23.7	15.1	28.4

**Table 4:** Data of each layer of atmospheric storage tanks.

Number of layers	1000m³		2000m³		3000m³		5000m³	
	Wall thickness (mm)	Floor height (m)	Wall thickness (mm)	Floor height (m)	Wall thickness (mm)	Floor height (m)	Wall thickness (mm)	Floor height (m)
Bottom	9	–	10	–	11	–	12	–
1st layer	8	1.8	9	1.8	10	1.8	11	1.8
2nd layer	8	1.8	8	1.8	9	1.8	10	1.8
3rd layer	8	1.8	8	1.8	8	1.8	9	1.8
4th layer	8	1.8	8	1.8	8	1.8	8	1.8
5th layer	8	1.8	8	1.8	8	1.8	8	1.8
6th layer	8	2.9	8	1.8	8	1.8	8	1.8
7th layer	–	–	8	2.3	8	1.8	8	1.8
8th layer	–	–	–	–	8	1.2	8	2.5
Roof	6	–	6	–	6	–	8	–

**Table 5:** Thermal physical properties of gasoline.

Combustion calorific value (kJ/kg)	Mass burning rate (kg/m²·s)	Density (kg/m^3^)	Specific heat (kJ/kg·°C)	Thermal conductivity (W/m·°C)	C/H ratio
44000	0.055	725	2.22	0.11	8:18

Under windless conditions:


$$\frac{H}{D}=\text{4}\text{2}{\left[\frac{m^{{}^{\prime \prime }}}{\rho_{\text{0}}\sqrt{gD}}\right]}^{\text{0}.\text{6}\text{1}}$$ (2-1)

Under windy conditions:


$$\frac{H}{D}=\text{5}\text{5}{\left(\frac{m^{*}}{\rho_{o}\sqrt{gD}}\right)}^{\text{0}.\text{6}\text{7}}\times {\left(u^{*}\right)}^{-\text{0}.\text{2}\text{1}}$$ (2-2)

$$\frac{H}{D}=\text{5}\text{5}{\left(\frac{m^{*}}{\rho_{o}\sqrt{gD}}\right)}^{\text{0}.\text{6}\text{7}}\times {\left(u^{*}\right)}^{-\text{0}.\text{2}\text{1}}$$ (2-3)

$$m^{n}=m^{*}\left(\text{1}-e^{-k\beta D}\right)$$ (2-4)

Where H denotes the flame height, m; $\rho_{o}$ is the air density, taken as 1.2 kg/m³; D represents the pool fire diameter, m; g is the gravitational acceleration, taken as 9.8 m/s²; $\;u^{*}$ denotes the wind speed, m/s; *u* is the wind speed at 10 m height, m/s. $\rho_{v}$ represents the vapor density of the flammable liquid, kg/m³. ${\text{m}}^{{}^{\prime \prime }}$ denotes the mass burning rate of the fuel, kg/m² ∙ s. $m^{*}$ is the maximum mass burning rate, kg/m² ∙ s. k denotes the flame attenuation coefficient, ${\text{m}}^{-\text{1}}$. β is the average beam length correction factor, where the kβ value for gasoline is 2.1 ${\text{m}}^{-\text{1}}$.

#### 2.1.2. Thermal response model.

The thermal radiation energy from the pool fire of an ignited storage tank to the target storage tank can be calculated using the Stefan-Boltzmann law [29], with the formula as follows:


I=EFat (2-5)

$$E=\varepsilon \sigma \left(T_{\text{1}}^{\text{4}}-T_{\text{0}}^{\text{4}}\right)$$ (2-6)

$$F=\sqrt{F_{H}^{\text{2}}+F_{V}^{\text{2}}}$$ (2-7)

$$F_{H}=\frac{\text{1}}{\pi }\left[\frac{\left(B-\frac{\text{1}}{S}\right)}{\sqrt{B^{\text{2}}-\text{1}}}{\text{tan}}^{-\text{1}}\left(\frac{\left(B+\text{1})(S-\text{1}\right)}{\left(B-\text{1})(S+\text{1}\right)}\right)-\frac{\left(A-\frac{\text{1}}{S}\right)}{\sqrt{A^{\text{2}}-\text{1}}}{\text{tan}}^{-\text{1}}\left(\frac{\left(A+\text{1})(S-\text{1}\right)}{\left(A-\text{1})(S+\text{1}\right)}\right)\right]$$ (2-8)

$$F_{V}=\frac{\text{1}}{\pi }\left[\frac{\text{1}}{S}{\text{tan}}^{-\text{1}}\frac{h}{\sqrt{S^{\text{2}}-\text{1}}}+\frac{h}{S}\left\{{\text{tan}}^{-\text{1}}\sqrt{\frac{S-\text{1}}{S+\text{1}}}-\frac{A}{\sqrt{A^{\text{2}}-\text{1}}}{\text{tan}}^{-\text{1}}\left[\frac{\left(A+\text{1})(S-\text{1}\right)}{\left(A-\text{1})(S+\text{1}\right)}\right]\right\}\right]$$ (2-9)

$$A=\frac{h^{\text{2}}+S^{\text{2}}+\text{1}}{\text{2}S}$$ (2-10)

$$B=\frac{\text{1}+S^{\text{2}}}{\text{2}S}$$ (2-11)

$$\tau =\left\{ \;\;\begin{array}{cc} \text{0}.\text{9}\text{7}\text{6}d^{-\text{0}.\text{0}\text{5}}, & d<\text{5}𝐦\\ \text{1}.\text{0}\text{2}\text{9}d^{-\text{0}.\text{0}\text{9}}, & \text{5}\leq d\leq \text{5}\text{5}𝐦\\ \text{1}.\text{1}\text{5}\text{9}d^{-\text{0}.\text{1}\text{2}}, & d>\text{5}\text{5}𝐦 \end{array}\right.\;$$ (2-12)

Where I refers to the thermal radiation intensity received by the target storage tank, kW/m². E denotes the thermal radiation intensity of the ignited storage tank, kW/m²; F is the view factor; α represents the surface absorptivity of the storage tank wall, with a value set at 0.6. $\sigma =\text{5}.\text{67}{\text{E}}^{-\text{8}}$W/(m^2^·K^4^*)*; τ stands for atmospheric transmittance; ε is the surface emissivity where ε = 1. $T_{\text{1}}$ indicates the flame temperature of the ignited storage tank, K. $T_{\text{0}}$ is the ambient temperature taken as 273 K; $F_{H}$ and $F_{V}$ are the horizontal and vertical view factors respectively. d refers to the horizontal distance, m. S is the ratio of the horizontal distance to the pool fire radius. *A* and *B* are the preset calculation coefficients; h is the height-to-diameter ratio of the storage tank.

**Fig 1:**
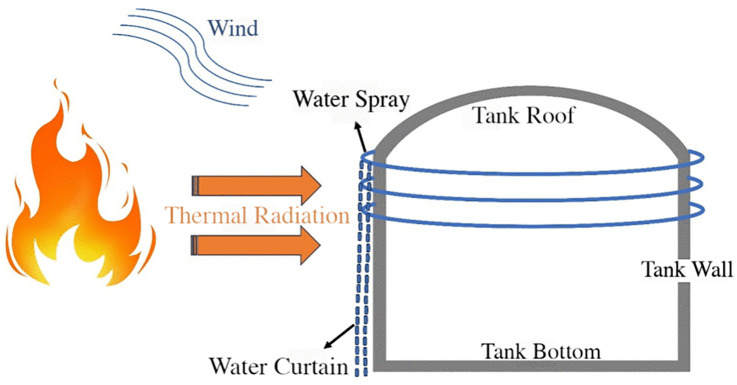
Schematic diagram of water spray system protection.

The water spray system forms a film of water on the surface of the storage tank, which weakens the thermal radiation from the flame through both blockage and absorption. This process slows down the failure of the target storage tank. Fig 1 provides a schematic diagram illustrating the protective effect of the water spray system during the tank’s thermal response process.

### 2.2. Establishment of numerical simulation scenarios

**Fig 2:**
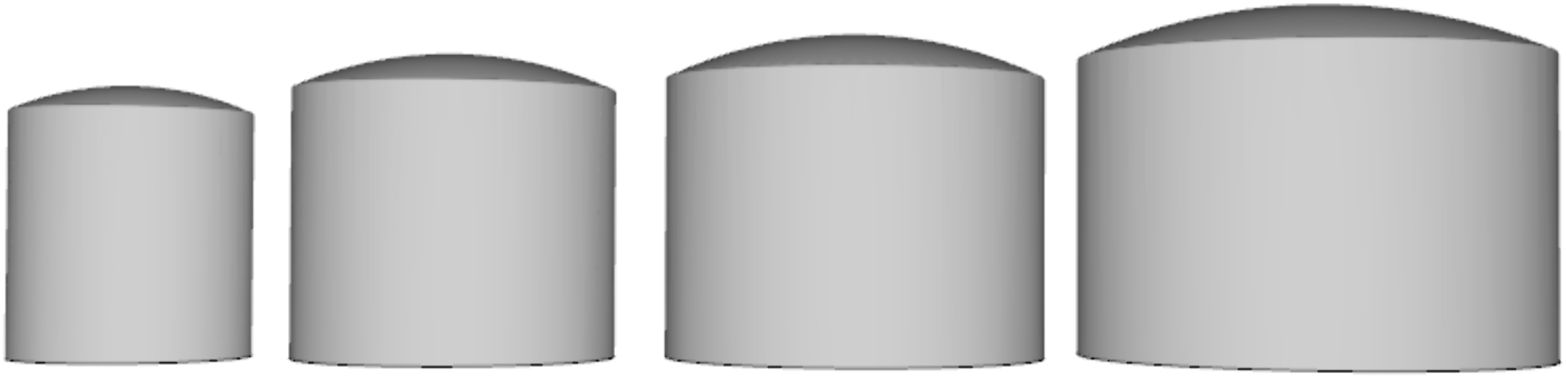
Pyrosim models of atmospheric storage tanks with different volumes.

In this study, fire separation distances of 0.4D, 0.6D, 0.8D, and 1.0D are adopted for simulation analysis, where D is the tank diameter, and 1.2 times the tank diameter is taken as the radius of curvature of the dome roof. The basic data of storage tanks with different volumes are listed in Table 3. Table 4 presents the wall thicknesses of each layer and height data of atmospheric storage tanks with different volumes. Fig 2 shows the Pyrosim models of storage tanks with different volumes.

**Fig 3:**
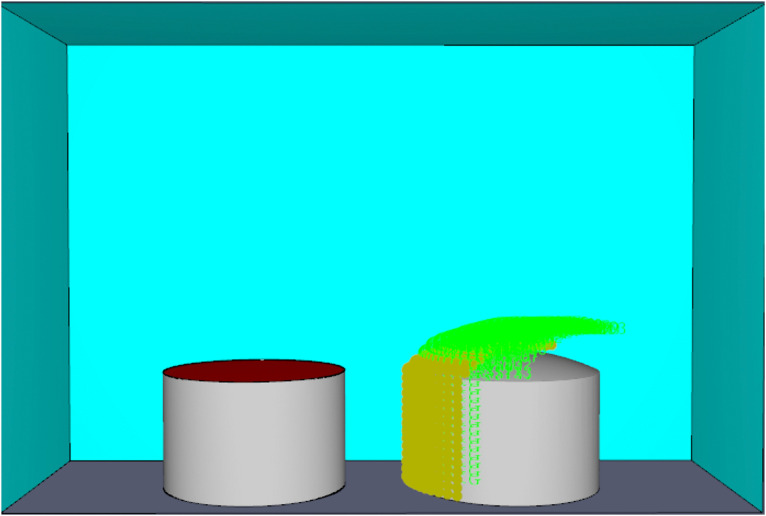
Atmospheric storage tank model in Pyrosim.

Gasoline was used as the combustion medium for the tank farm fire, with specific thermophysical parameters detailed in Table 5. The top and sides of the model’s spatial domain were set to OPEN conditions to allow the free flow of combustion products and air, thereby representing the open atmosphere of an outdoor tank farm. The ground was set to INERT to simulate a non-combustible surface, preventing additional heat feedback from ground burning. The ambient temperature was fixed at 20°C to reflect standard environmental conditions commonly used in pool fire simulations. These boundary settings are consistent with previous studies on large-scale tank fire simulations and ensure both physical realism and computational stability. Based on the grid sizes set by previous scholars [30,31], this study selected a cubic grid size of 0.5 m × 0.5 m × 0.5 m for the atmospheric storage tank pool fire simulation, with the simulation time set to 100 s. The simulation model established using Pyrosim and the distribution of thermal radiation flux detection points are shown in Fig 3.

**Fig 4:**
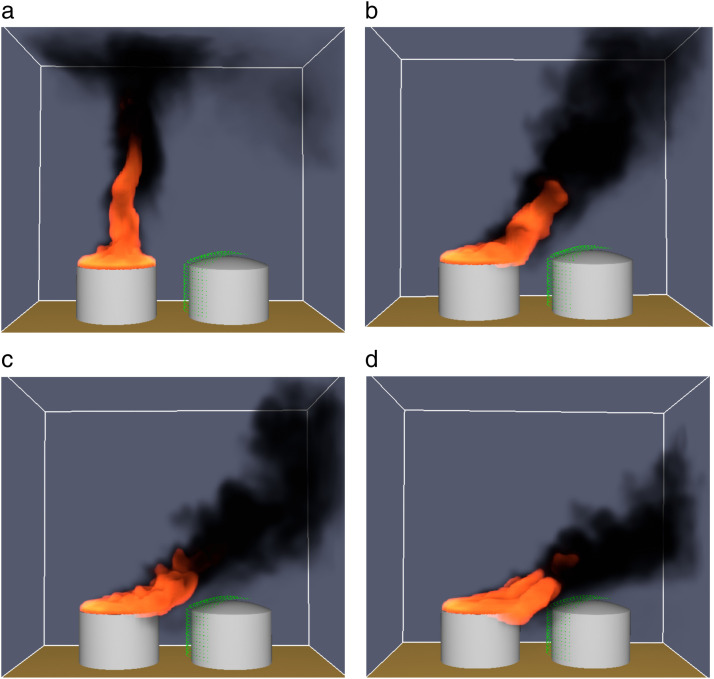
Flame model of a 5000m³ storage tank under different wind speeds. **(a) Wind speed of 2m/s, (b) Wind speed of 4m/s**, **(c) Wind speed of 6m/s**, **(d) Wind speed of 8m/s.**

The main model and working condition parameters are summarized in Table 6. To ensure numerical accuracy, a grid independence test was performed by comparing results obtained with grid sizes of 0.6 m, 0.5 m, and 0.4 m. The variation in predicted thermal radiation flux was less than 2% when refining the grid from 0.5 m to 0.4 m, indicating that the chosen grid size of 0.5 m × 0.5 m × 0.5 m provides sufficient accuracy while maintaining computational efficiency. The pool fire flame models of the 5000 m³ storage tank under different wind speeds are shown in Fig 4. To validate the simulation model, the calculated flame height and thermal radiation values were compared with the empirical correlations proposed by Thomas [27] and Babrauskas [28]. The deviations were within 8%, which is consistent with previous validation studies, confirming that the model is reliable for thermal radiation prediction.

**Table 6:** Summary of simulation model parameters and operating conditions.

Tank volume(m^3^)	1000	2000	3000	5000
**Fire separation distance(m)**	0.4D, 0.6D, 0.8D, 1.0D			
**Ambient wind speed(m/s)**	0, 2, 4, 6, 8			
**Grid size(m**^**3**^)	0.5 × 0.5 × 0.5			
**Computational domain(m**^**3**^)	40 × 15 × 40	55 × 20 × 40	65 × 25 × 50	80 × 30 × 60

In this study, a 5000 m³ vertical atmospheric storage tank was selected as the representative case because it corresponds to a commonly used capacity for large gasoline storage tanks in Chinese chemical industrial parks. This capacity also falls within the high-risk category identified in national safety regulations, making it representative for domino accident analysis.The fire separation distance was set to 0.4D (where D is the tank diameter), in accordance with the Technical Specification for Petroleum Storage (GB 50074–2014). This distance is frequently adopted in quantitative risk assessment studies and represents the minimum regulatory requirement under Chinese design standards. By using this distance, the study not only ensures practical relevance but also evaluates safety barrier performance under the most conservative conditions.

The pool fire flame models of the 5000 m³ storage tank under different wind speeds are shown in Fig 4. As wind speed increases, the flame tilt angle becomes more pronounced, and the flame length is elongated in the downwind direction. This enhances the thermal radiation received by the target tank on the downwind side, while reducing radiation to the upwind side. Specifically, under calm conditions (0 m/s), the flame plume rises vertically, and the radiation distribution is relatively uniform around the tank. At higher wind speeds (e.g., 6–8 m/s), the flame bends significantly, leading to asymmetric heat transfer and concentrated thermal impact on adjacent tanks in the downwind direction. These results are consistent with findings from previous studies, confirming that wind is a critical factor in fire propagation and safety barrier performance.

## 3. Simulation results

### 3.1. Numerical simulation of thermal radiation effect on storage tanks under the influence of ambient wind without a water spray system

#### 3.1.1. Analysis of the temperature field of the target storage tank.

**Fig 5:**
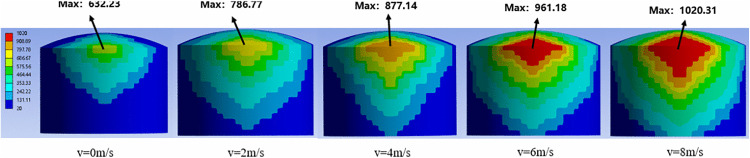
Temperature field distribution of 5000m³ tanks under different wind speeds.

In this section, taking the thermal response process of the 5000 m³ atmospheric storage tank under a fire separation distance of 0.4D as an example, the temperature field distribution on the tank surface under different wind speed conditions is analyzed, and the simulation results are shown in Fig 5.

From the temperature distribution on the tank wall, it can be seen that the maximum temperature is mainly distributed at the connection between the side (facing the flame) and the top of the storage tank. Moreover, as the distance increases, the temperature gradually decreases from the top to the bottom of the tank, and also gradually decreases from the central axis to both sides, showing an axisymmetric distribution.

**Fig 6:**
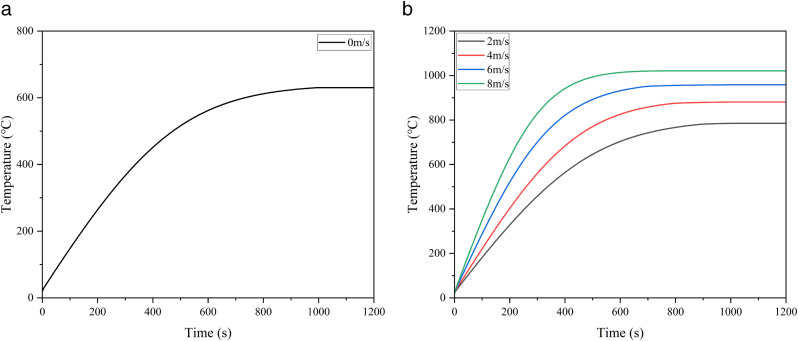
Temporal evolution of the maximum wall temperature in 5000 m³ tanks. (a) Variation of maximum temperature under windless conditions, (b) Variation of maximum temperature under different wind speed conditions.

The curve of temperature variation with time at the maximum temperature location is shown in Fig 6. It can be seen from the figure that the temperature rise rate of the storage tank when there is wind is significantly faster than that when there is no wind. The greater the wind speed, the larger the thermal radiation load, the faster the temperature rises, and the shorter the time to reach the peak. When there is no wind, the temperature rise rate of the storage tank slows down after approximately 700 s and reaches the peak at around 950 s; under a wind speed of 8 m/s, the temperature rise rate slows down at about 400 s and reaches the peak at around 600 s. After the heat exchange between the tank wall and the pool fire reaches thermal equilibrium, the temperature tends to be stable.

#### 3.1.2. Analysis of the stress field of the target storage tank.

**Fig 7:**
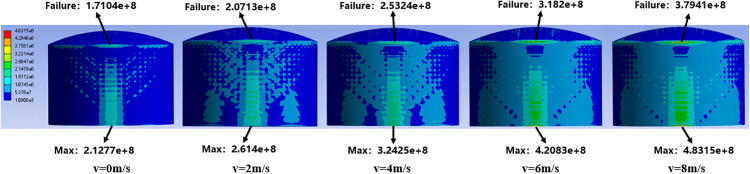
Stress field distribution of 5000m³ tanks under different wind speeds.

In this section, taking the 5000 m^3^ atmospheric storage tank with a fire separation distance of 0.4D as an example, the thermal radiation effect under the action of ambient wind at 0–8 m/s is studied. Fig 7 shows the numerical simulation results of stress and strain on the tank surface.

It can be seen from the figure that the maximum Mises stress of the tank wall is located at the connection between the bottommost circle of the tank wall and the tank bottom, and it decreases from bottom to top along the height of the tank wall. An increase in wind speed leads to an increase in thermal radiation intensity, resulting in an increase in the Mises stress on the tank surface and a significant expansion of its distribution area.

**Fig 8:**
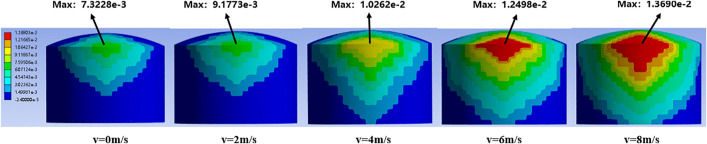
Variation of thermal strain in 5000 m³ tanks under varying wind speeds.

Fig 8 shows the thermal strain distribution diagram of the atmospheric storage tank under different wind speeds. When the wind speed reaches 8 m/s, the maximum thermal strain generated on the tank wall is 0.01369 m, while it is only 0.0073228 m under windless conditions.

#### 3.1.3. Establishment of the failure time model for the target storage tank.

**Fig 9:**
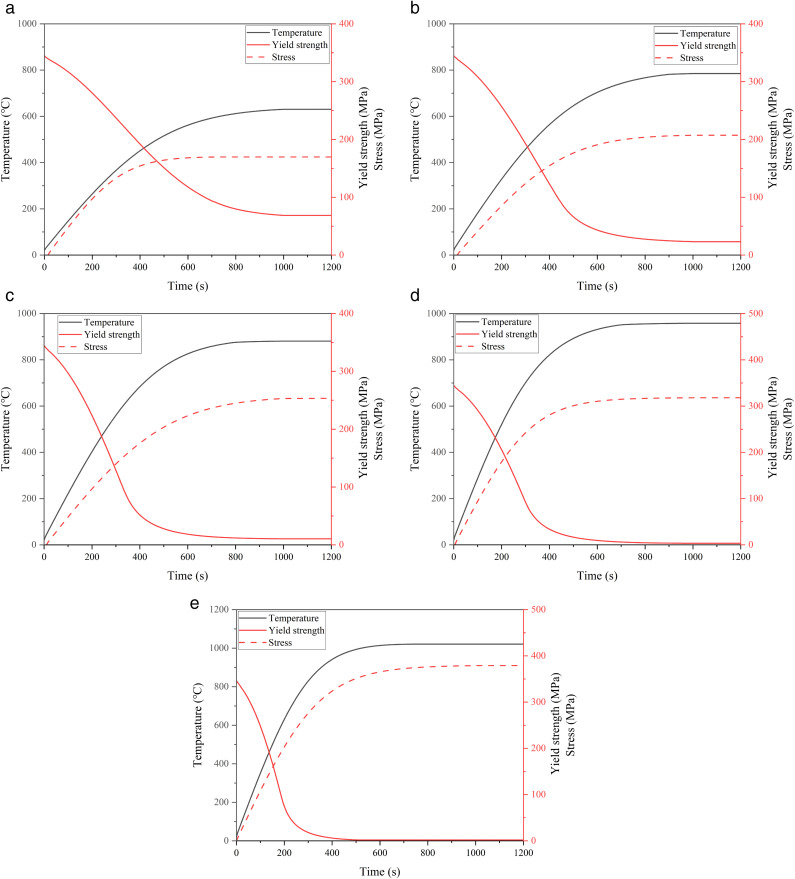
Temperature, yield strength, and stress variation curves for the failure section of 5000m³ tanks under different wind speed conditions. (a) Variation curve at 0m/s, (b) Variation curve at 2m/s, (c) Variation curve at 4 m/s, (d) Variation curve at 6 m/s, (e) Variation curve at 0 m/s.

In existing studies on the variation of steel yield strength with temperature, the relational expression proposed by the European Convention for Construction of Steel (ECCS) based on experimental data [32] is relatively conservative and widely applied. Therefore, this paper uses this relational expression and, by analyzing the results of relevant variables in the simulation, draws the curves of temperature, stress, and yield strength changes at the location with the highest temperature (failure point) on the wall of the 5000 m³ target storage tank under the thermal radiation of the ignited storage tank under ambient wind conditions of 0 m/s, 2 m/s, 4 m/s, 6 m/s, and 8 m/s, as shown in Fig 9.

It can be seen from the intersection position of yield strength and stress in the figure that the failure times of the storage tank under wind speeds of 0 m/s, 2 m/s, 4 m/s, 6 m/s and 8 m/s are approximately 465 s, 374 s, 290 s, 206 s and 149 s, respectively.

**Fig 10:**
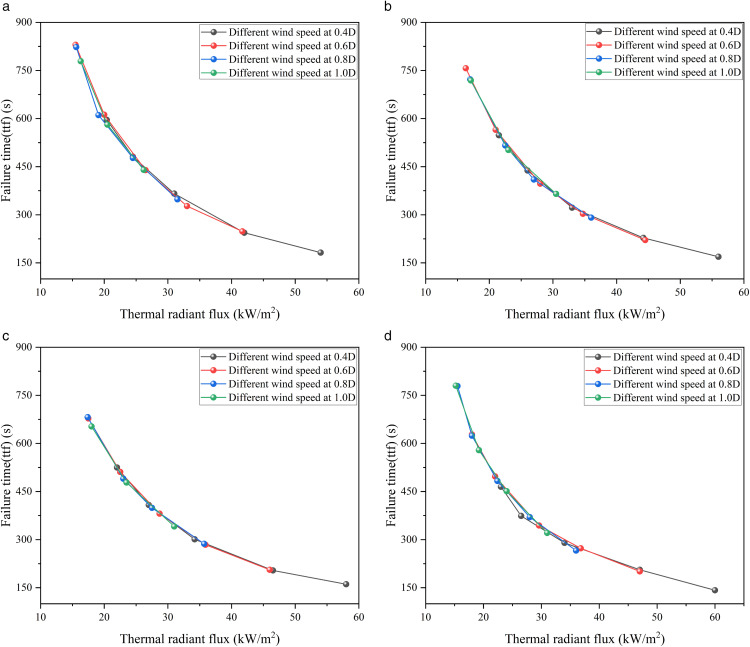
The time to failure of target tank under various wind speeds and separations. (a) Failure time curve of 1000m³ tanks, (b) Failure time curve of 2000m³ tanks, (c) Failure time curve of 3000m³ tanks, (d) Failure time curve of 5000m³ tanks.

Based on the specific values of the temperature field, stress field and failure time of the above-mentioned storage tanks, the failure time curves of 1000 m³, 2000 m³, 3000 m³ and 5000 m³ atmospheric storage tanks are plotted, as shown in Fig 10.

Fig 10 illustrates the time to failure (TTF) of the target tank under different wind speeds and with/without the water deluge system. It can be observed that in the absence of protection, the TTF decreases sharply with increasing wind speed, dropping from 465 s at 0 m/s to 149 s at 8 m/s. With the deluge system in place, the TTF is significantly prolonged, although the beneficial effect diminishes as wind speed increases. This indicates that while the deluge system is effective in delaying failure, high wind conditions considerably weaken its protective capacity. These results highlight the critical role of environmental factors in determining safety barrier performance.

Using the formula proposed by Cozzani and Landucci [33], the tank failure time obtained from the simulation is fitted to establish a failure time criterion model for atmospheric storage tanks under the action of pool fire thermal radiation, as shown in Equation 3-1 (with a goodness of fit of 0.9879).


$$\text{ln}\left(ttf_{\text{1}}\right)=-\text{1}.\text{2}\text{0}\text{0}\text{6}\text{ln}\left(I_{\text{1}}\right)-\text{2}.\text{3}\text{6}\text{9}\text{6}\times {\text{1}\text{0}}^{-\text{5}}V+\text{1}\text{0}.\text{0}\text{2}\text{5}\text{8}$$ (3-1)

Where ${ttf}_{\text{1}}$ represents the failure time of the target storage tank under the action of pool fire thermal radiation, s. $I_{\text{1}}$ is the thermal radiation value received by the target storage tank without the protection of a water spray system, kW/m². V denotes the volume of the target storage tank, m³. For validation, the TTF values were also computed using the empirical correlation proposed by Cozzani [33]. The comparison showed that the simulation results deviate by less than 10% from the empirical formula predictions across the considered wind speed range. This agreement demonstrates that the numerical model adopted in this study is consistent with established methodologies and reliable for performance evaluation.

### 3.2. Numerical simulation on thermal radiation effect of storage tank under the influence of ambient wind with water spray system

In accordance with the research objectives of this chapter, the structure and triggering mechanism of the water spray device are simplified to improve simulation efficiency. When the water spray system of the target storage tank detects a fire, it is activated immediately. A water curtain is formed through spraying to block and absorb the thermal radiation from the pool fire, thereby protecting the storage tank.

In this study, the protective effect is described by introducing a variable called the thermal radiation attenuation coefficient, denoted by α. The calculation formula of the attenuation coefficient α is as follows:


$$\alpha =\frac{\Delta I}{I_{\text{1}}}=\frac{I_{\text{1}}-I_{\text{2}}}{I_{\text{1}}}$$ (3-2)

Equation (3-2) is inspired from recent TTF prediction models such as those proposed by Amin, Li Mo, and Yang et al. [34–36], which establish failure time estimation under pool fire considering structural response, coupling effects, and environmental factors. Using these as benchmarks ensures that our model is aligned with state-of-the-art methods. Where $I_{\text{1}}$ is the thermal radiation received by the target storage tank without the protection of the water spray system, kW/m². $I_{\text{2}}$ is the thermal radiation received by the target storage tank with the protection of the water spray system, kW/m².

#### 3.2.1. Simulation scenario of the storage tank with water spray system.

**Fig 11:**
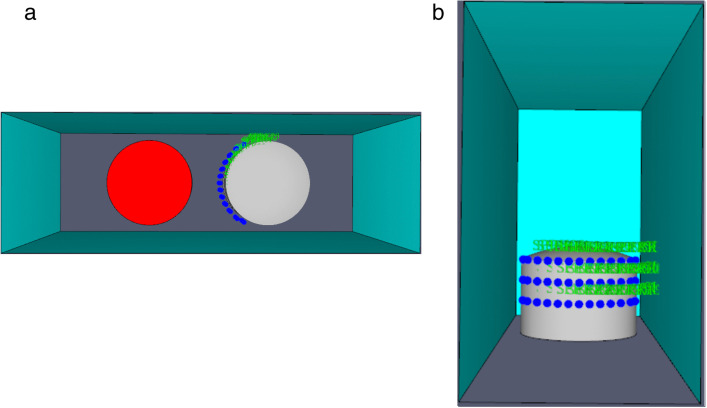
The 5000m³ atmospheric tank model under the effect of water spray system. (a) The simulation model diagram of a 5000m³ tank, (b) Water spray model.

In this study, water spray system are installed at a lateral spacing of 2 m on the side close to the accident storage tank. The devices are arranged in three circles (upper, middle, and lower), with 13 devices in each layer, totaling 39 devices. The model diagram is shown in Fig 11, and the relevant parameters of the water spray system are listed in Table 7.

**Table 7:** Water spray system parameters for a 5000m³ atmospheric tank.

Name	Upper cooling pipe	Middle cooling pipe	Lower cooling pipe
**Height (m)**	15.1	11.0	7.0
**Number of sprinklers**	13	13	13
**Water system pressure (MPa)**	0.7 ~ 1.2		
**Water supply intensity (L/(min ∙ m** ^**2**^ **))**	2, 5, 10, 15, 20		

The thermal radiation flux detectors are arranged on the same side as the water spray system and slightly close to the wall of the target storage tank, with an interval of approximately 1 m between each detector.

#### 3.2.2. Analysis of the influence of water spray intensity on protection effect.

**Fig 12:**
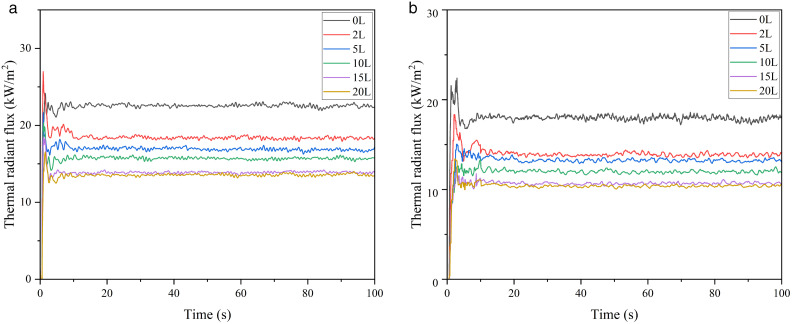
Thermal radiation for 5000m³ tanks under different water spray intensities. (a) Thermal radiation under 0.4D separation, (b) Thermal radiation under 0.6D separation.

In this section, by studying the thermal radiation of the 5000 m³ atmospheric storage tank under the fire separation distances of 0.4D and 0.6D, the influence degree of water spray intensity on the weakening effect of the thermal radiation received by the target storage tank is explored, as shown in Fig 12.

It can be seen from the figure that as the water spray intensity increases, the thermal radiation value on the tank surface decreases under the same fire separation distance. When the intensity is 2 L/(min·m²), the thermal radiation flux is reduced by approximately 20%. When the intensity reaches 15–20 L/(min·m²), the change in the thermal radiation value on the target tank surface slows down. Considering the comprehensive cost, the water spray intensity of 15 L/(min·m²) has the optimal efficiency. Therefore, this intensity is used as the main water supply intensity in the subsequent sections of this paper.

#### 3.2.3. Analysis of the influence of fire separation distance on the protection effect of water spray system.

**Fig 13:**
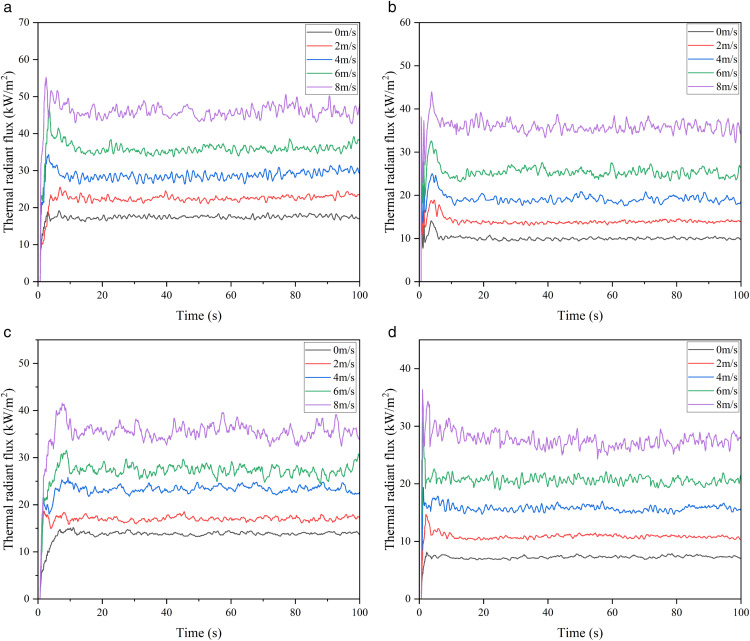
Thermal radiation flux on the wall of 3000 m³ tanks under different separation and protection conditions. (a) Thermal radiation at 0.6D separation without water spray, (b) Thermal radiation at 0.6D separation under water spray, (c) Thermal radiation at 0.8D separation without water spray, (d) Thermal radiation at 0.8D separation under water spray.

Taking the 3000 m³ atmospheric storage tank with fire separation distances of 0.6D and 0.8D as an example, the variation of thermal radiation value and attenuation coefficient with distance is studied, and the simulation results are shown in Fig 13.

**Fig 14:**
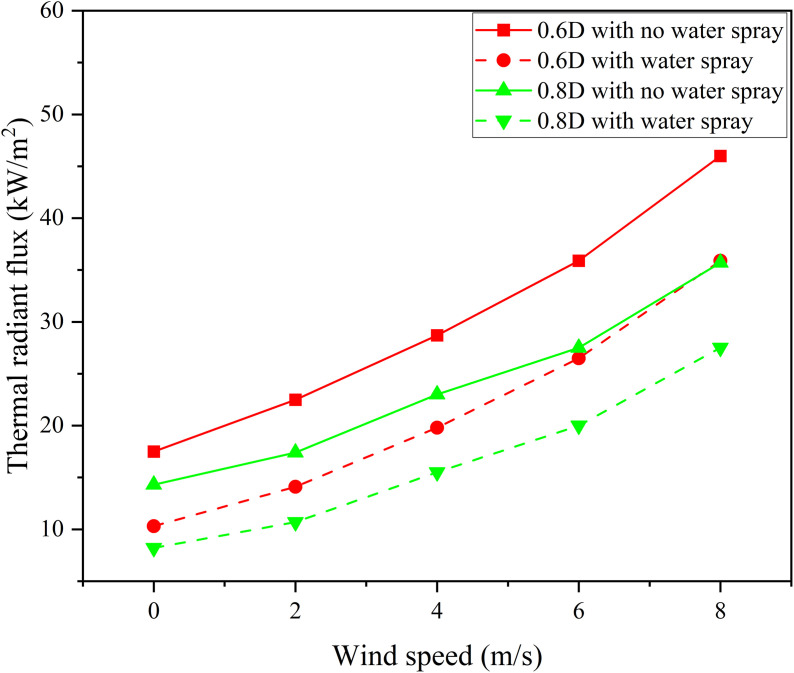
Thermal radiation of tanks with and without water spray under different separations.

**Fig 15:**
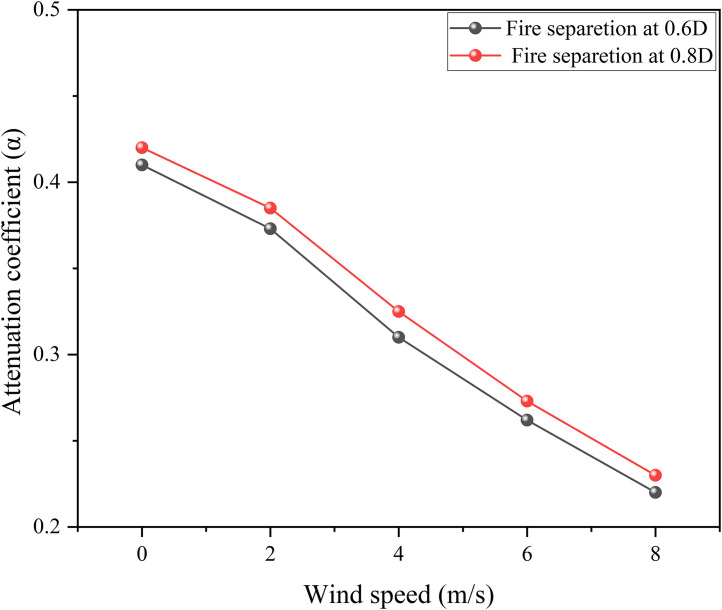
Thermal radiation attenuation coefficient of water spray under different separations.

The results show that: there is a positive correlation between wind speed and thermal radiation value, and the greater the wind speed, the more significant the fluctuation of thermal radiation monitoring value; as the fire separation distance increases, the thermal radiation value decreases. The water spray system can effectively weaken the thermal radiation intensity and delay the failure of the storage tank. After data processing, the thermal radiation values and weakening coefficients under different working conditions are detailed in Fig 14,15.

Fig 14 shows that the water spray system significantly weakens thermal radiation. Under the fire separation distance of 0.6D, when the wind speed is 8 m/s, the thermal radiation value on the tank surface decreases from 46 kW/m² to 35.9 kW/m², with an attenuation coefficient of approximately 0.22. Fig 15 indicates that an increase in wind speed leads to a decrease in the attenuation coefficient of the water spray system, while an increase in fire separation distance causes it to increase. However, in comparison, the influence of wind speed on the protection effect is far greater than that of fire separation distance. Considering simulation errors, this study selects a fire separation distance of 0.4D and focuses on studying the influence of ambient wind speed on the protection effect and attenuation coefficient of the water spray system.

#### 3.2.4. Analysis of the influence of wind speed on the protection effect of the water spray system.

**Fig 16:**
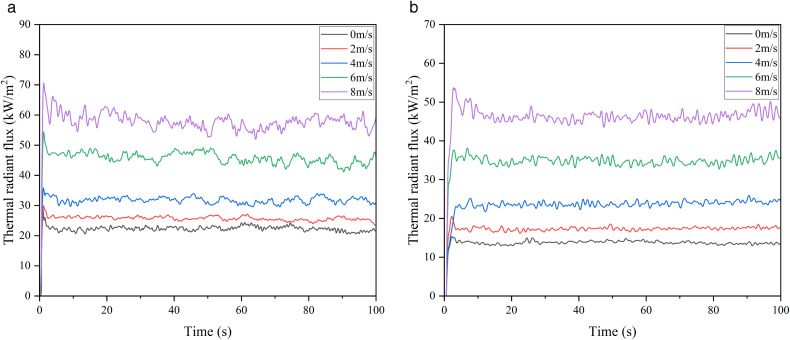
Thermal radiation flux on the wall of 5000 m³ tanks under different wind speeds and protection conditions. (a) Thermal radiation under different wind speeds without water spray, (b) Thermal radiation under different wind speeds with water spray.

**Fig 17:**
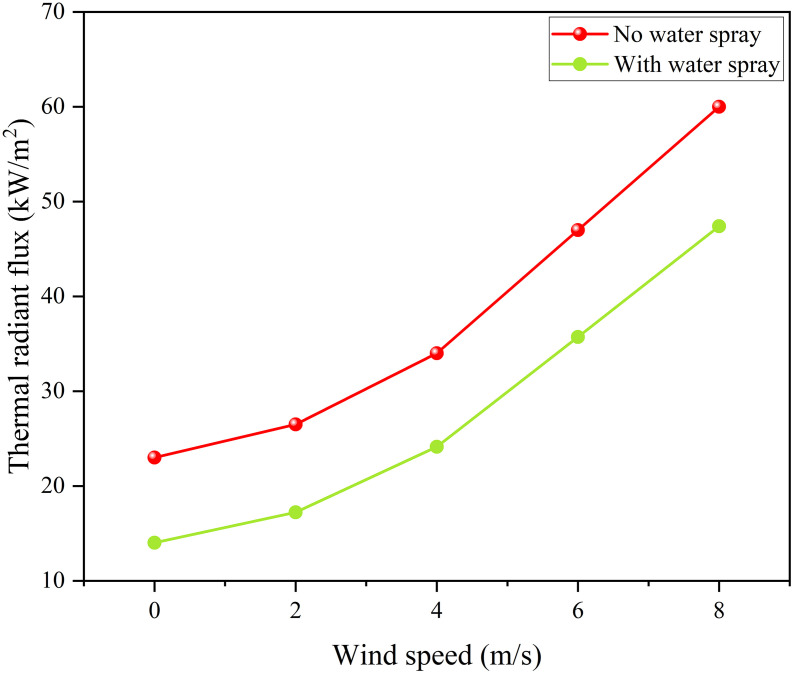
Thermal radiation for tanks under different wind speeds.

**Fig 18:**
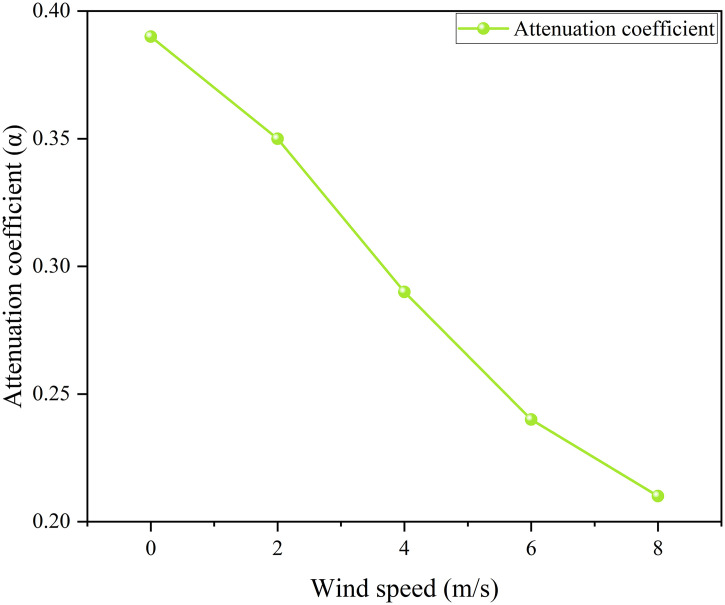
Thermal radiation attenuation by water spray under different wind speeds.

In this section, taking the 5000 m³ atmospheric storage tank as an example, the influence of wind speed on the attenuation coefficient of the water spray system under a fire separation distance of 0.4D is analyzed, and the simulation results are shown in Fig 16–18.

It can be seen from Fig 16,17 that the ambient wind significantly increases the thermal radiation intensity of the target storage tank: when the wind speed is 0 m/s without a water spray system, the thermal radiation value is 23 kW/m², and it rises to 60 kW/m² at a wind speed of 8 m/s, with the protection of the water spray system, the thermal radiation value drops to 14 kW/m² at a wind speed of 0 m/s and 47.4 kW/m² at a wind speed of 8 m/s. The attenuation coefficients calculated under different wind speeds are shown in Fig 18, where the attenuation coefficient decreases as the wind speed increases: it is 0.39 at 0 m/s and drops to 0.21 at 8 m/s. The relational expression between the attenuation coefficient and wind speed is obtained by fitting (Equation 3-2, with a goodness of fit of 0.993). These results demonstrate that wind speed significantly reduces the protective performance of the water spray system, especially under high-wind conditions. As the attenuation coefficient directly affects the thermal radiation received by the target tank, it is essential to integrate this coefficient into the prediction of tank failure time. Therefore, the next subsection develops a failure time model based on the above attenuation behavior.


$$a=\text{0}.\text{0}\text{0}\text{0}\text{5}\text{4}\nu^{\text{2}}-\text{0}.\text{0}\text{2}\text{7}\text{8}\nu +\text{0}.\text{3}\text{9}\text{4}\text{3}$$ (3-3)

Where α is the attenuation coefficient of the water spray system. α is the ambient wind speed, m/s.

Therefore, the pool fire thermal radiation received by the target storage tank under the protection of the water spray system can be expressed as:


$$I_{\text{2}}=(\text{1}-a)I_{\text{1}}$$ (3-4)

$I_{\text{1}}$ is the thermal radiation value received by the target storage tank without the protection of the water spray system, kW/m². $I_{\text{2}}$ is the thermal radiation value received by the target storage tank with the protection of the water spray system, kW/m².

#### 3.2.5. Establishment of the failure time model for atmospheric storage tanks under the water spray system.

Substituting Equation 3-4 into the tank failure time model under thermal radiation, it serves as the failure time model of the storage tank under the protection of the water spray system, as shown in Equation 3-5:


$$\text{ln}\left(ttf_{\text{2}}\right)=-\text{1}.\text{2}\text{0}\text{0}\text{6}\text{ln}\left[(\text{1}-\alpha )I_{\text{1}}\right]-\text{2}.\text{3}\text{6}\text{9}\text{6}\times {\text{1}\text{0}}^{-\text{5}}V+\text{1}\text{0}.\text{0}\text{2}\text{5}\text{8}$$ (3-5)

Where ${ttf}_{\text{2}}$ is the failure time of the storage tank under the water spray system, seconds s. α is the attenuation coefficient of the water spray system. $I_{\text{1}}$ is the thermal radiation value received by the target storage tank, kW/m². V is the volume of the atmospheric storage tank, m³.

Based on the simulation results, the failure time of the atmospheric storage tank under the protection of the water deluge system was determined for different wind speeds. The TTF decreased from 715 s at 0 m/s to 326 s at 8 m/s, compared to 465 s and 149 s in the unprotected case. This indicates that the water deluge system can significantly delay tank failure, although its effectiveness decreases with increasing wind speed.

A regression model was established to represent the failure time as a function of wind speed (v):


$${\;\text{TTF}}_{\text{deluge}}=\text{a}-\text{bv}\;({\text{R}}^{\text{2}}=\text{0}.\text{97})$$ (3-6)

where a and b are fitting parameters derived from the simulation data. This model provides a practical tool for estimating the protective performance of the water deluge system under varying environmental conditions.

## 4. Performance evaluation of typical automatic technical safety barriers based on bayesian networks

In this chapter, a method for calculating the effectiveness and reliability of the water spray system is established using Bayesian networks [35]. Combined with these two indicators, a quantitative performance evaluation model for the water spray system is constructed, providing a quantitative method for the quantitative expression of the protection effect of the water spray system in chemical storage tank areas.

The extension ratio of failure time E₁ and the reduction ratio of failure probability E₂ are calculated as the effectiveness indicators of the water spray system, where E represents the effectiveness indicator. The main calculation formulas are as follows:


$$E_{\text{1}}=\frac{ttf_{\text{1}}-ttf_{\text{2}}}{ttf_{\text{1}}}=\frac{\Delta TTF}{ttf_{\text{1}}}$$ (4-1)

$$E_{\text{2}}=\frac{P_{\text{1}}-P_{\text{2}}}{P_{\text{1}}}$$ (4-2)

$$E=\frac{E_{\text{1}}+E_{\text{2}}}{\text{2}}$$ (4-3)

Where ${ttf}_{\text{1}}\;\text{and}\;{ttf}_{\text{2}}$ are the failure times of the target storage tank without and with the water spray system, respectively, s. ΔTTF is the extended failure time of the storage tank by the water spray system, in seconds s. $P_{\text{1}}$ is the failure probability of the target storage tank without the water spray system. $P_{\text{2}}$ is the failure probability of the target storage tank with the water spray system.

Most existing safety barrier performance assessments rely on the ARAMIS framework, which evaluates effectiveness, response time, and the resulting confidence level. However, this framework’s analysis of effectiveness often falls short by neglecting the dynamic nature of real-world protection, making accurate quantitative assessment of control effects difficult. Additionally, response time is highly uncertain, influenced by environmental and human factors. Consequently, the ARAMIS system is considered a semi-quantitative method due to its lack of quantitative data support, dependence on expert judgment, and limited adaptability to dynamic risks.

To overcome these limitations, this study has developed a new approach. Through extensive data simulations, we’ve quantitatively calculated safety barrier effectiveness. This was then combined with Bayesian network probabilistic reasoning to determine barrier reliability. By integrating effectiveness and reliability, we derived a performance score, enabling a precise quantitative assessment. This method allows for a more accurate comparison of different barrier performances.

### 4.1. Case study

In this section, the atmospheric tank farm of an oil depot belonging to a chemical storage enterprise in a certain location is selected as the research object. There are 6 storage tanks in this tank farm, all of which are of the same size, with a volume of 5000 m³, an outer diameter of 23.7 m, and a tank height of 15.1 m. The internal storage medium is gasoline, with a filling coefficient of 75% and a storage capacity of 2719 t. The material of the tank wall is Q345 steel.

The thermal radiation intensity received by each storage tank in the tank farm under the action of the pool fire from the accident tank is obtained by simulating and analyzing the consequences of the tank pool fire accident using ALOHA software, as shown in Table 8.

**Table 8:** Thermal radiation received by the target tanks from the accident tank.

Accident tank	Target tank thermal radiation(kW/m^2^)					
	T_1_	T_2_	T_3_	T_4_	T_5_	T_6_
**T**_**1**_	—	23.6	15.8	14.7	4.9	3.6
**T**_**2**_	14.2	—	13.5	15.8	4.2	4.9
**T**_**3**_	15.8	14.7	—	23.6	15.8	14.7
**T**_**4**_	13.5	15.8	14.2	—	13.5	15.8
**T**_**5**_	4.9	3.6	15.8	14.7	—	23.6
**T**_**6**_	4.2	4.9	13.5	15.8	14.2	—

**Fig 19:**
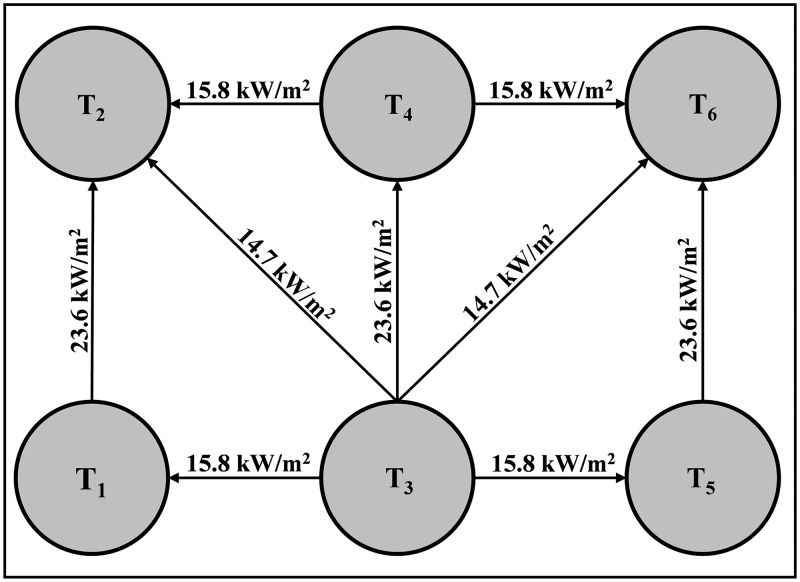
Accident propagation path (no water spray system).

**Fig 20:**
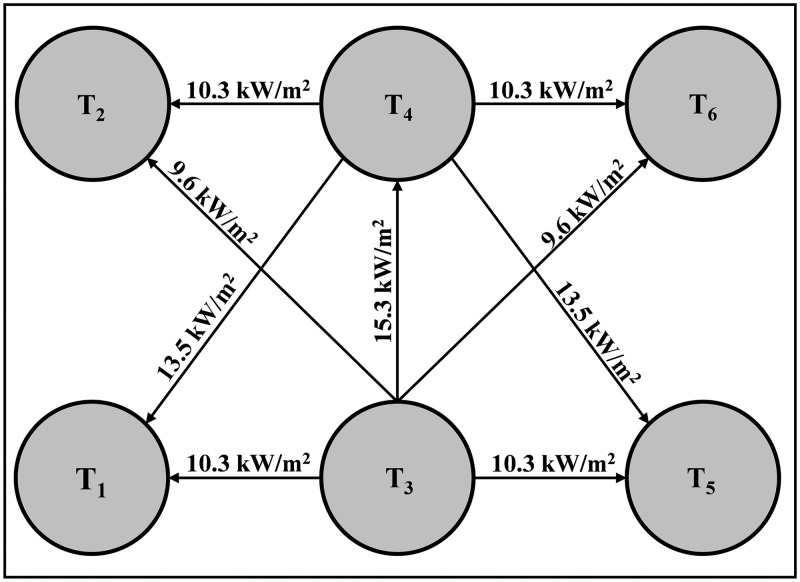
Accident propagation path (with water spray system).

Tank T3 is selected as the initial accident tank because it is located at the center of the storage tank group and contains gasoline, which is identified as the most frequent hazardous substance initiating domino accidents (23.9% of cases). Its spatial position also places it in close proximity to multiple adjacent tanks, making it more likely to trigger cascading effects. Therefore, T3 represents a conservative and representative scenario for evaluating domino accident propagation. The specific accident propagation path in the tank farm without the protection of water spray systems is: T_3_ → T_1_ + T_4_ + T_5_ → T_2_ + T_6_, as shown in Fig 19. The specific accident propagation path in the tank farm with the protection of water spray systems is: T_3_ → T_4_ → T_1_ + T_2_ + T_5_ + T_6_, as shown in Fig 20.

### 4.2. Calculation of the effectiveness of the water spray system

**Fig 21:**
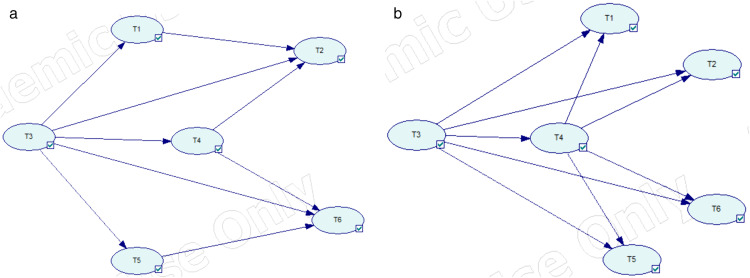
Bayesian network models for the tank area. **(a) No water spray system**, **(b) With water spray system.**

A static Bayesian network model for the tank farm is constructed using GeNIe Academic 5.0 software. The failure probabilities of each storage tank with and without the water spray system, calculated respectively, are shown in Table 9. The Bayesian network model of the tank farm is presented in Fig 21.

**Table 9:** Failure probability of storage tanks in the tank area.

Tank code	Failure probability	
	Without water spray system	With water spray system
T_1_	1.7549 × 10^−6^	1.1652 × 10^−8^
T_2_	1.0071 × 10^−5^	4.6641 × 10^−9^
T_4_	8.8866 × 10^−6^	1.2399 × 10^−6^
T_5_	1.7549 × 10^−6^	1.1652 × 10^−8^
T_6_	1.0339 × 10^−5^	4.6641 × 10^−9^

Analysis of the above table shows that the water spray system can significantly reduce the failure probability of storage tanks. Taking tanks T3 and T4 as examples for evaluation, substituting the failure probability data of tank T4 into Formula 4−2 gives E₂ = 0.86, and the final calculated effectiveness index of the water spray system is E = 0.77.

### 4.3. Calculation of the reliability of the water spray system

The formula for calculating the failure probability of the water spray system is shown in Equation 4-4.


R(t)=1−Q(t) (4-4)

Where Q(t) is the failure probability of the water spray system. R(t) denotes the reliability probability of the water spray system.

Table 10 shows the probabilities of each node event obtained from the literature review statistics. The probabilities of root node events were obtained from reliability studies of automatic water spray systems [37,38] and statistical failure data reported in industrial reliability databases. These studies are widely used in quantitative risk assessment of fire protection systems, providing reliable input parameters for Bayesian analysis [39–41].

**Table 10:** Probability of occurrence of root node events.

Root node	Probability
Water source drying up	0.00001
Water pump failure	0.017
Main power supply failure	0.055
Standby power supply failure	0.0001
Controller failure	0.0008
Detector failure	0.0001
Sprinkler failure	0.00001
Pipeline failure	0.0015
Valve failure	0.0042

**Fig 22:**
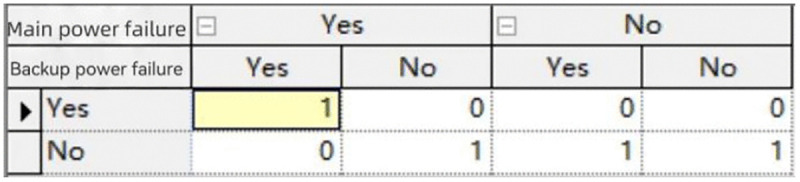
Conditional probability table for the “Power Supply Failure” node.

**Fig 23:**
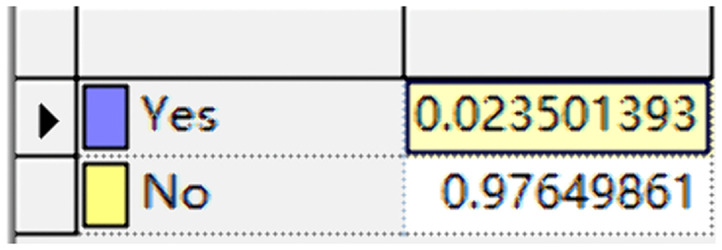
Failure probability of the water spray system.

The data in the above table are defined in the conditional probability tables of the Bayesian network model. These values are widely used in reliability analyses of water spray systems and provide a statistically valid basis for Bayesian network modeling. The conditional probability tables of some nodes and the failure probability of the water spray system are shown in Fig 22,23, respectively. For example, pump failure probability (0.017) was derived from historical maintenance data of similar fire water supply systems, while power supply failure probabilities (0.055 for main and 0.0001 for backup) were based on large-scale reliability surveys. These values reflect realistic failure frequencies and have been applied in prior risk assessment research.

The Bayesian network model was developed to quantify the reliability of the water deluge system. The model structure is shown in Table 10, where the root nodes represent component-level failure events (e.g., pump failure, valve failure, power supply failure), and intermediate nodes represent subsystem performance. The system failure is defined as the top event.

The failure probabilities of root nodes were obtained from published reliability studies and industrial reliability databases. For example, pump failure probability was derived from historical maintenance records of fire water supply systems, while power supply failure probabilities were obtained from large-scale reliability surveys.

The conditional probability tables (CPTs) were constructed by defining logical dependencies between nodes. For instance, the success of the pump subsystem requires both the pump and the power supply to be operational. If either fails, the subsystem is considered failed. In such cases, CPT entries were assigned deterministically (e.g., P[subsystem failure | pump failure] = 1). For probabilistic dependencies, literature-based failure rates were used to calculate the conditional probabilities.

Where direct data were unavailable, reasonable assumptions were made based on expert judgment, consistent with prior Bayesian reliability modeling studies. A sensitivity analysis was performed by varying root node probabilities by ±20%, and the system reliability R(t) varied by less than 5%, confirming the robustness of the results.

To identify the components that most strongly influence the overall reliability, a one-at-a-time sensitivity analysis was performed on the Bayesian network. In this approach, the prior failure probability of each component node was individually perturbed within a small range (±10%), while all other nodes were kept constant. The resulting change in the posterior failure probability of the top event (i.e., system failure) was then recorded. The magnitude of this change was used as the sensitivity index, allowing the identification of the most critical nodes within the system.

What’s more, the sensitivity analysis was conducted to identify which root nodes have the greatest impact on the system reliability R(t). The results show that pump failure and main power supply failure are the most influential contributors to system reliability variation. When their failure probabilities were increased by 20%, the overall system reliability decreased by 3.1% and 2.4%, respectively. In contrast, the effects of other root nodes (e.g., detector failure, sprinkler failure, water source failure) were below 1%. This indicates that the water pump subsystem and power supply subsystem are the critical weak links in the water spray system.

The failure probability of the water spray system, calculated by running GeNIe Academic 5.0 software, is Q(t) ≈ 0.0235. Meanwhile, the reliability probability of the system, R(t) = 0.9765, is derived through calculation using Formula 4−4.

### 4.4. Performance evaluation of the water spray system

The performance evaluation model of the water spray system based on effectiveness and reliability is as follows [20]:


$$P_{total}=E\cdot R(t)$$ (4-5)

Where $P_{total}$ is the performance score of the water spray system. R(t) is the reliability probability of the water spray system.

The effectiveness evaluation index E and reliability probability R(t) of the water spray system are obtained through calculations using numerical simulation results and Bayesian network tools. Substituting the data into Formula 4–5, the performance score of the water spray system is calculated as $P_{total}$ = 0.77 × 0.9765 ≈ 0.752. To evaluate robustness, a sensitivity analysis was performed by varying each root node probability by ±20%. The resulting system reliability R(t) changed by less than 5%, indicating that the final performance score ($P_{total}$ ≈ 0.752) is not highly sensitive to minor fluctuations in input probabilities.

## 5. Discussion

Our results demonstrated that tank failure time decreased sharply with wind speed, while the water deluge system substantially delayed failure, though with reduced effectiveness at higher wind speeds. This confirms the strong influence of environmental conditions on barrier performance.

These findings are consistent with earlier studies showing that wind conditions significantly intensify thermal radiation and reduce the efficiency of water-based protective systems [26]. Moreover, the observed failure time reduction agrees with the thermal response patterns reported by Zhou et al. [14] and Ding et al. [15]. Compared with Landucci et al. [16], who evaluated barrier performance mainly through semi-quantitative methods, our work further extends the literature by integrating simulation-based effectiveness with Bayesian reliability analysis. Compared with ARAMIS, which relies primarily on qualitative judgments, our quantitative approach provides more precise predictions of barrier performance. Recent advances using dynamic Bayesian networks also emphasize the importance of reliability-based assessment, which our study integrates with effectiveness analysis.

The developed performance index ($P_{total}$= 0.752) provides a quantitative benchmark for evaluating water-based protective barriers in storage tank farms.The method can support risk-informed decision-making, such as determining minimum fire separation distances or prioritizing maintenance of critical components (e.g., pumps, power supply). The simulations considered a specific 5000 m³ gasoline tank, which may limit generalizability to other tank sizes or substances.The Bayesian probabilities were partly based on literature data rather than site-specific statistics. The sensitivity analysis of the Bayesian network further indicates that the most critical weak links of the water spray system are the pump failure node and the main power supply failure node, which contribute the largest share of variation to the system reliability. This highlights that maintenance priority should focus on pump subsystem health and ensuring stable power supply, as these components most significantly influence the overall reliability of the barrier system.

This study is limited by its reliance on numerical simulations and by the assumption of idealized water spray system configurations. Experimental validation and consideration of aging, clogging, or dynamic degradation of components were beyond the present scope but warrant further investigation.

Despite these limitations, the study contributes a quantitative framework that integrates environmental attenuation, thermal-response modeling, and Bayesian reliability analysis—offering a more comprehensive and transferable method for evaluating safety barrier performance. The findings also provide practical insights into how environmental wind and system-component reliability influence protective effectiveness in tank farms. Future research should extend the model to dynamic accident scenarios, incorporate other barrier types (e.g., foam systems, emergency shut-down devices), and validate against large-scale experimental data.

## 6. Conclusions

This study establishes a quantitative framework for evaluating the performance of water spray systems in atmospheric chemical storage tank areas by integrating simulation-based effectiveness analysis with Bayesian network–based reliability assessment. Unlike traditional semi-quantitative approaches such as ARAMIS, the proposed framework explicitly incorporates environmental conditions, thermal-response modeling, and system-level probability calculations, thus offering a more comprehensive basis for barrier evaluation.

The results demonstrate that water spray systems substantially mitigate thermal radiation impact and delay tank failure under pool-fire conditions, confirming their essential role in preventing domino effects. However, the protective performance is highly sensitive to wind conditions, emphasizing the need to consider environmental variability in design and operation. The reliability analysis further identifies critical nodes—particularly pumps and power supply—that most strongly influence system performance, providing practical guidance for targeted maintenance and risk-control strategies.

Overall, this work contributes a more rigorous and transferable methodology for evaluating safety-barrier performance in chemical storage facilities. The framework can support risk-informed decision-making and can be extended in future research to other types of active and passive barriers or validated using experimental and real-incident data.

## Supporting information

S1 Data Minimum data set. (RAR)
